# Sociodemographic variation in symptomatic faecal immunochemical testing return: a population-based analysis of 125 659 patients

**DOI:** 10.3399/BJGP.2025.0144

**Published:** 2026-01-27

**Authors:** Emily Haworth, Caroline Addison, Willie Hamilton, Colin Rees, Ian Dunn, Pete Wheatstone, Linda Sharp, Christina Dobson

**Affiliations:** 1 Population Health Sciences Institute, Newcastle University, Newcastle upon Tyne, UK; 2 Gateshead Health NHS Foundation Trust, Newcastle upon Tyne, UK; 3 Medical School, University of Exeter, Exeter, UK; 4 South Tyneside and Sunderland NHS Foundation Trust, South Shields, UK; 5 Newcastle University, Newcastle upon Tyne, UK

**Keywords:** bowel cancer, early diagnosis, faecal immunochemical testing, health inequalities, primary health care, test completion

## Abstract

**Background:**

In the UK, patients presenting in primary care with possible symptoms of colorectal cancer complete faecal immunochemical testing (FIT) as triage for (urgent) colorectal investigation. Little is known about FIT completion rates or sociodemographic variations in these.

**Aim:**

To measure overall FIT return for the year 2023 and assess sociodemographic variation in completion.

**Design and setting:**

A population-based analysis of FIT requests made in 2023 to one pathology laboratory serving the North East, North Yorkshire, and Shropshire.

**Method:**

The study included patients aged ≥18 years, for whom sex, postcode sector, and return status were recorded. Index of Multiple Deprivation quintiles, ethnicity tertiles, and rural–urban categories were assigned. Multiple logistic regression assessed associations between sociodemographic characteristics and test (non-)return within 10 weeks. Sensitivity analyses were undertaken: a) excluding younger patients (aged <50 years); and b) removing the 10-week window for test return.

**Results:**

In total, 93% (*n* = 116 786/125 659) of patients returned their test. Of those who returned them, 54% (*n* = 63 534) did so within 1 week; only 5% (*n* = 5637) took >3 weeks. Patients aged <50 years, male patients, those in the most deprived and ethnically diverse areas, and urban residents all had a significantly higher likelihood of non-return. Findings were unchanged in sensitivity analyses.

**Conclusion:**

Although FIT completion was high, sociodemographic patterning of (non-)return was evident. Further work is needed on barriers to and facilitators of FIT completion to inform measures to address these observed inequalities and support patients to access timely diagnosis.

## How this fits in

Symptomatic faecal immunochemical testing (FIT) was introduced to prioritise patients at greatest risk of colorectal cancer for urgent investigation, while simultaneously managing endoscopy resources. Little is known, to date, about overall test completion or variation in completion, and therefore the potential consequences of this new pathway for colorectal cancer inequalities. Analysis of symptomatic FIT requests for 2023 made to the Gateshead pathology hub showed that symptomatic FIT return was high, at 93% (*n* = 116 786/125 659); however, people in the most deprived areas, most ethnically diverse areas, males, and younger patients were significantly less likely to return tests. Further work is required to understand and address these disparities to minimise any potential exacerbation of cancer inequalities.

## Introduction

Colorectal cancer (CRC) is the fourth most common cancer in the UK, accounting for >44 000 new cases and >16 000 deaths each year.^1^ Most CRCs are diagnosed after patients present in primary care with symptoms, such as altered bowel habit, rectal bleeding, unexplained weight loss, and abdominal/rectal masses.^2^

Faecal immunochemical testing (FIT) is now a core step in the referral of patients presenting with symptoms of possible CRC. FIT detects and measures occult blood in the stool, with concentrations predictive of CRC risk. In the UK, adults with concentrations ≥10 µg Hb/g should be referred on a suspected cancer pathway.^3,4^ The test entails patients collecting a small sample of stool at home, then posting it to the designated pathology laboratory. Symptomatic FIT appears to be acceptable to patients, who see it as a mechanism to be diagnosed earlier or address their symptoms.^5^ Patient satisfaction with the process of completing symptomatic FIT also appears to be generally high.^6^

FIT does, however, introduce an additional ‘task’ in the pathway of symptomatic patients, which ultimately gatekeeps access to secondary care. If a patient does not return their test, their progress along the investigative pathway may be delayed; this could have significant consequences for time to diagnosis and outcomes.^7^ Concerns among healthcare practitioners about rates of non-return are beginning to arise.^8^ There is some evidence to suggest that there may be sociodemographic patterning in symptomatic FIT return,^9^ which raises concerns that symptomatic FIT may introduce, compound, or widen inequalities. However, these data were from 2017 to 2021, when FIT was primarily used in low-risk patients. As of 2022, the British Society of Gastroenterology (BSG)/Association of Coloproctology of Great Britain and Ireland (ACPGBI) guidelines^10^ stated that FIT should be used for all patients (not just low risk) and it is vital that variation in current symptomatic FIT pathways are examined.

Inequalities in CRC diagnosis and outcomes have been well documented. Patients from more socioeconomically deprived areas,^11–13^ ethnically minoritised patients,^14^ and older patients^13,15^ all have poorer survival from CRC. It is important to understand whether there are:

- differences in how completion of the test varies by different patient groups;
- what causes these differences; and
- what they mean for CRC inequalities.

In the current article, analyses of symptomatic FIT requests made to one pathology laboratory over a 12-month period are presented along with an exploration of the patterns and variation in return by sociodemographic characteristics.

## Method

### Population

The Queen Elizabeth Hospital (QEH) Gateshead Pathology Laboratory is the sole laboratory that processes symptomatic FIT requests for NHS patients in the North East of England (Northumberland, Tyne & Wear, County Durham, and Teesside), parts of North Yorkshire, and parts of Shropshire. GPs make electronic requests to QEH, who post kits directly to patients (second class Royal Mail) and monitor and process returns. Kits (Mast Group) are prelabelled with patient information and include written and pictorial instructions on collection procedure. Patients collect a small, single sample of stool, write the collection date on the tube, then return it using the prepaid postage (second class) return envelope.

**Figure 1.**
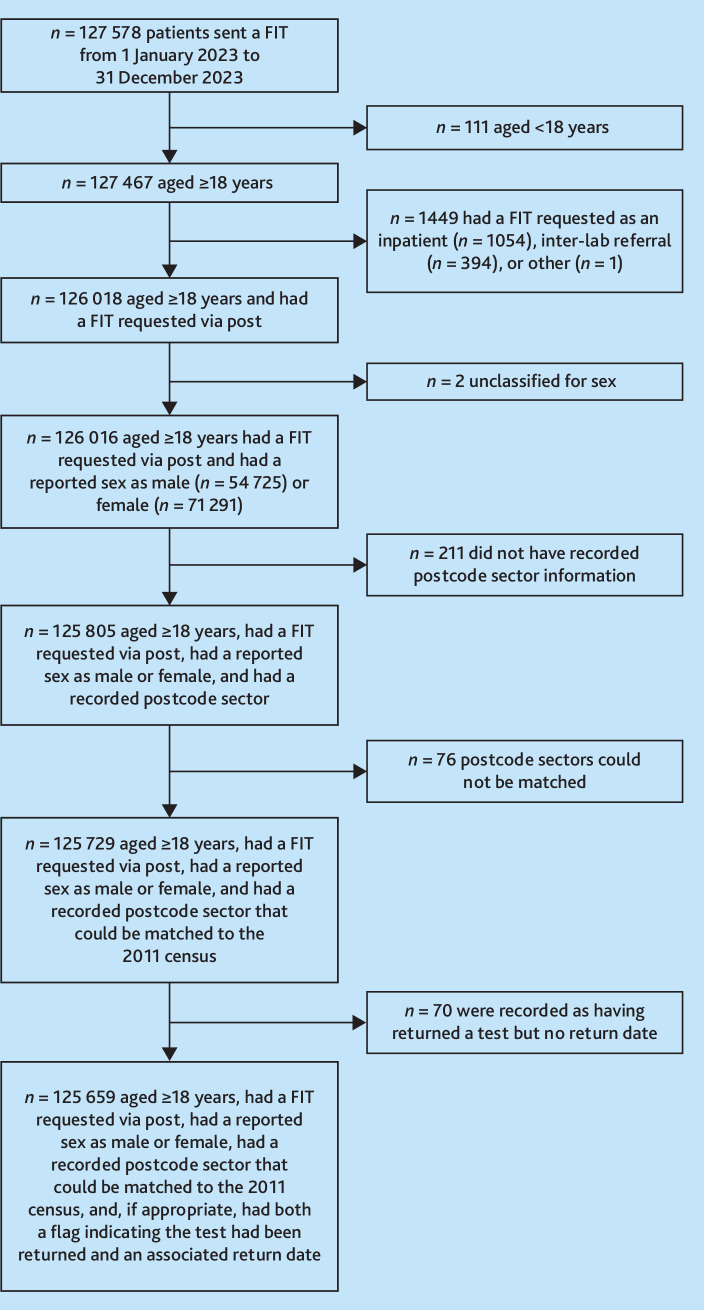
Consort diagram of inclusion criteria. FIT = faecal immunochemical test.

Records of symptomatic FIT requests made to the QEH between 1 January 2023 and 31 December 2023 were examined. The data were extracted on 21 March 2024, at which point all patients had been followed up for test completion for ≥10 weeks. The pseudonymised dataset included patient ID number (allocated at QEH from NHS number) and test-level data on FIT completion (date test requested and, if relevant, completed), postcode sector of residence, age, and sex. For the analysis, patients aged ≥18 years, who had a FIT requested by post (that is, from primary care), a record of their sex (male or female), a complete postcode sector (as identified in GeoConvert),^16^ and, if they had returned a FIT, a recorded return date were included (Figure 1). After the exclusion criteria were applied, the analysis dataset included 125 659 patients.

### Data analysis

All data were downloaded into Stata (version 18.5) for analysis.

Index of Multiple Deprivation^17^ (IMD) quintile, rural–urban classification, and ethnic diversity of patients’ area of residence were determined from their postcode sector. Using GeoConvert, the lower layer super output areas (LSOAs) within each postcode sector were identified. IMD quintile, rural–urban classification, and percentage of individuals of ethnicity other than white resident in each LSOA were abstracted from 2011 census data. Using weights based on LSOA populations, weighted IMD quintile (1 = least deprived; 5 = most deprived), weighted rural–urban category,^18^ and weighted ethnic diversity (percentage of individuals of ethnicity other than white) were computed for each postcode sector and assigned to the patients in the dataset. Ethnic diversity was collapsed into approximate tertiles for analysis (least ethnically diverse = ≤2.692%; moderately = 2.693%–4.693%; and most ethnically diverse = ≥4.694%). Patients’ age was categorised into four groups: 18–49; 50–64; 65–74; and ≥75 years. Rural–urban categories were collapsed into rural and urban for analysis.

The binary outcome variable for the primary analysis was FIT return (yes or no) within 10 weeks of the first test request date. Some patients were sent >1 FIT during 2023 (for example, GP submitted multiple requests). For analysis, test return at a patient level was considered, including the first and (if relevant) second tests for an individual. If the individual returned one test within 10 weeks, the information from that test (irrespective of whether it was the first or second) was used; if they returned both or neither within 10 weeks, the information from test one was used (see Supplementary Figure S1).

Chi-square tests of associations were conducted between fixed characteristics and FIT return. A multiple logistic regression was carried out to assess the relationships between age group, sex, IMD quintiles, rural–urban category, and ethnic diversity tertiles and the likelihood of not returning the FIT. The final multivariate model had adequate fit on the Hosmer–Lemeshow test.

Sensitivity analyses were carried out, rerunning the logistic regression: a) excluding those aged <50 years; and b) removing the 10-week return window cut-off and instead defining FIT completion based on whether patients had returned either their first or second kit at any point before the data extraction date (irrespective of how long it took them to return it). The number of returns by month was also documented to assess variation across the year. Information on time taken to return completed tests and numbers of patients who were sent multiple tests was summarised.

## Results

In 2023 the QEH dispatched symptomatic FIT to 127 578 patients. After exclusions, FIT was dispatched for 125 659 patients at the request of primary care, with the average number of requests per month being 10 472 (range 7744–11 758). Demographic characteristics of these patients are presented in Table 1.

**Table 1.** Demographic information for individuals to whom a symptomatic FIT was dispatched at the request of primary care (*N* = 125 659)

Characteristic	Patients, *n* (%)
**Age category, years**	
18–49	25 274 (20)
50–64	37 940 (30)
65–74	27 611 (22)
≥75	34 834 (28)
**Sex**	
Female	71 092 (57)
Male	54 567 (43)
**IMD quintile**	
1 (least deprived)	26 546 (21)
2	26 133 (21)
3	22 287 (18)
4	23 703 (19)
5 (most deprived)	26 990 (21)
**Rural category** ^a^	
Urban	96 376 (77)
Rural	29 283 (23)
**Ethnic diversity tertiles** ^b^	
1	45 915 (37)
2	40 266 (32)
3	39 478 (31)

a Urban included: city and town; city and town in a sparse setting; minor conurbation; and major conurbation. Rural included: hamlets and isolated dwellings; hamlets and isolated dwellings in a sparse setting; village; village in a sparse setting; town and fringe; and town and fringe in a sparse setting.

b Ethnicity tertiles: 1 = least ethnically diverse (≤2.692% ethnicity other than white); 2 = moderately ethnically diverse (2.693%–4.693% ethnicity other than white); and 3 = most ethnically diverse (≥4.694% ethnicity other than white). FIT = faecal immunochemical test. IMD = Index of Multiple Deprivation.

The majority of tests (80%, *n* = 100 385) were requested for those aged ≥50 years, with a mean patient age of 63 (standard deviation 15.6) years. More tests were requested for female patients than male patients (57%, *n* = 71 092 versus 43%, *n* = 54 567) and for those residing in urban, compared with rural, areas (77%, *n* = 96 376 versus 23%, *n* = 29 283). There was little difference in numbers of symptomatic FIT requests made by IMD quintiles and ethnic diversity tertiles (Table 1).

In the primary analysis, of the 125 659 individuals requested to complete symptomatic FIT by primary care in 2023, 116 786 (93%) did so (Table 2). Slightly more than half of those who returned a test within 10 weeks did so within 1 week of the kit request by primary care (54%, *n* = 63 534), 33% were returned between 1 and 2 weeks of the kit request (*n* = 38 413), 8% between 2 and 3 weeks (*n* = 9202), and 5% (*n* = 5637) were returned >3 weeks after the request. Of the 116 786 patients who returned a test, 1779 of these tests (2% of the overall sample) were ‘spoiled’ and not able to be processed.

**Table 2.** Associations between fixed characteristics and FIT returned either within 2 weeks or >2 weeks (*N* = 116 786)

Characteristic	Returned within 2 weeks, *n* (%),*N* = 101 947	Returned over 2 weeks, *n* (%),*N* = 14 839	χ^2^	*P*-value
**Age category, years**			2200.00	<0.001
18–49	17 697 (17)	4621 (31)		
50–64	30 586 (30)	5094 (34)		
65–74	24 089 (24)	2282 (15)		
≥75	29 575 (29)	2842 (19)		
**Sex**			1.96	0.161
Female	57 851 (57)	8511 (57)		
Male	44 096 (43)	6328 (43)		
**IMD quintiles**			110.90	<0.001
1 (least deprived)	22 101 (22)	3085 (21)		
2	21 753 (21)	2854 (19)		
3	18 148 (18)	2632 (18)		
4	19 204 (19)	2726 (18)		
5 (most deprived)	20 741 (20)	3542 (24)		
**Rural category** ^**a**^			28.14	<0.001
Urban	77 586 (76)	11 587 (78)		
Rural	24 361 (24)	3252 (22)		
**Ethnic diversity tertiles** ^**b**^			80.16	<0.001
1	37 707 (37)	5183 (35)		
2	33 101 (32)	4584 (31)		
3	31 139 (31)	5072 (34)		

^a^Urban included: city and town; city and town in a sparse setting; minor conurbation; and major conurbation. Rural included: hamlets and isolated dwellings; hamlets and isolated dwellings in a sparse setting; village; village in a sparse setting; town and fringe; and town and fringe in a sparse setting. ^b^Ethnicity tertiles: 1 = least ethnically diverse (≤2.692% ethnicity other than white); 2 = moderately ethnically diverse (2.693%-4.693% ethnicity other than white); and 3 = most ethnically diverse (≥4.694% ethnicity other than white). FIT = faecal immunochemical test. IMD = Index of Multiple Deprivation.

Age, deprivation, rural–urban residence, and ethnicity tertiles were all significantly associated with the time to return FIT (Table 2). Younger patients, those residing in the most deprived areas, in the most ethnically diverse areas, and in urban areas were all more likely to take ≥3 weeks to return their FIT. Sex was not associated with timeliness of return.

In univariate tests, all fixed characteristics were significantly associated with return status (Table 3). Non-return was higher in those aged <50 years (12%, *n* = 2956/25 274), those living in the most deprived areas (10%, *n* = 2707/26 990), and those living in the most ethnically diverse areas (8%, *n* = 3267/39 478).

**Table 3.** Associations between fixed characteristics and FIT return status in primary analysis: numbers and percentages, χ^2^ statistics and *P*-values, multivariable odds ratios for non-return, with 95% confidence intervals, and likelihood ratio test *P*-values

Variable	Non-return, *n* (%),*N* = 8873^a^	Return, *n* (%),*N* = 116 786^a^	Univariate	Multivariable			
			*P*-value^b^	OR	95% CI	*P*-value	LRT *P*-value
**Age category, years**			<0.001				<0.001
18–49	2956 (12)	22 318 (88)		2.04	1.93 to 2.16	<0.001	
50–64	2260 (6)	35 680 (94)		1		–	
65–74	1240 (4)	26 371 (96)		0.75	0.70 to 0.81	<0.001	
≥75	2417 (7)	32 417 (93)		1.23	1.15 to 1.30	<0.001	
**Sex**			<0.001				<0.001
Female	4730 (7)	66 362 (93)		1		–	
Male	4143 (8)	50 424 (92)		1.17	1.12 to 1.22	<0.001	
**IMD quintiles**			<0.001				<0.001
1 (least deprived)	1360 (5)	25 186 (95)		1		–	
2	1526 (6)	24 607 (94)		1.20	1.11 to 1.30	<0.001	
3	1507 (7)	20 780 (93)		1.37	1.27 to 1.48	<0.001	
4	1773 (7)	21 930 (93)		1.51	1.40 to 1.63	<0.001	
5 (most deprived)	2707 (10)	24 283 (90)		1.95	1.82 to 2.10	<0.001	
**Rural category^c^**			<0.001				0.029
Urban	7203 (7)	89 173 (93)		1		–	
Rural	1670 (6)	27 613 (94)		0.94	0.88 to 0.99	0.030	
**Ethnicity diversity tertiles^d^**			<0.001				<0.001
1	3025 (7)	42 890 (93)		1		–	
2	2581 (6)	37 685 (94)		1.04	0.99 to 1.10	0.137	
3	3267 (8)	36 211 (92)		1.19	1.13 to 1.26	<0.001	

^a^Percentages are calculated at row percentage. ^b^ *P*-value corresponds to the χ^2^-test statistic. ^c^Urban included: city and town; city and town in a sparse setting; minor conurbation; major conurbation. rural included; hamlets and isolated dwellings; hamlets and isolated dwellings in a sparse setting; village; village in a sparse setting; town and fringe; town and fringe in a sparse setting. ^d^Ethnicity tertiles: 1 = least ethnically diverse (≤2.692% ethnicity other than white), 2 = moderately ethnically diverse (2.693–4.693% ethnicity other than white) and 3 = most ethnically diverse (≥4.694% ethnicity other than white). FIT = faecal immunochemical test. IMD = Index of Multiple Deprivation. LRT = likelihood ratio test. OR = odds ratio.

Table 3 shows the results of multiple logistic regression analyses. All variables made a statistically significant contribution to the model. After adjusting for other variables, those aged <50 years were twice as likely to not return a FIT compared with those aged 50–64 years (multivariable odds ratio [OR] 2.04, 95% confidence interval [CI] = 1.93 to 2.16). Likelihood of non-return increased with increasing deprivation (IMD quintile 5 versus IMD quintile 1: multivariable OR 1.95, 95% CI = 1.82 to 2.10). Similarly, non-return increased with increasing ethnic diversity (most versus least diverse: multivariable OR =1.19, 95% CI = 1.13 to 1.26). Male patients were 17% more likely than females to not return a FIT. Non-return was less common among rural than urban residents (multivariable OR 0.94, 95% CI = 0.88 to 0.99).

Both sensitivity analyses (excluding those aged <50 years and then removing the 10-week return window) revealed similar patterns and magnitude of effect estimates for non-return (see Supplementary Tables S1 and S2).

## Discussion

### Summary

Overall, return of symptomatic FIT was high, with only 7% (*n* = 8873) of patients not returning a test. Return was associated with deprivation, with people from more deprived areas having significantly lower test return rates. Patients residing in the most ethnically diverse areas also had lower return rates and were more likely to take >3 weeks to return their test. Non-return of FIT was also found to be significantly more likely in males and younger patients.

### Strengths and limitations

Key strengths of this study are the large sample size and the inclusion of a complete 12 months of FIT requests. In addition, because the QEH is the single laboratory providing and analysing FIT requests from the NHS in North East England, as well as bounded parts of North Yorkshire and Shropshire, this is in effect a population-based analysis, thus minimising selection bias.

The study analysed 2023 data because the authors of the current study judged that the BSG/ACPGBI guidelines should be embedded in primary care pathways by this point. However, analysing only 1 year of data precluded examination of temporal trends in symptomatic FIT completion. This could provide further insight into return patterns and direct future intervention focus.

The QEH laboratory serves a large population that includes areas with notable socioeconomic disparities.^19^ Therefore, these findings should have reasonable generalisability to other regions. However, ethnic diversity is lower than average, and the current study’s analysis of variation in completion by ethnicity was based on an LSOA-derived proxy measure of ethnic mix. Therefore, the magnitude of inequalities pertaining to ethnicity may have been underestimated. It is important that future work examines individual-level ethnicity, where available, while also taking an intersectional approach to understanding the multiple factors that may influence test completion.

### Comparison with existing literature

This analysis showed a return rate of 93% (*n* = 116 786) for symptomatic FIT, higher than reported in other areas (for example, 88% in Scotland^20^ and 90%–91% in Nottingham^9^). It is also notably higher than has been assumed for symptomatic FIT, with qualitative interviews with pathology services highlighting concerns about non-return rates of up to 20%.^21^ The analyses in the current study suggest that such concerns may have been built on consideration of return at the kit level (where individuals who had received multiple kits are in effect double counted) as opposed to patient level, as has been used in the current study. There is also variation in FIT distribution processes across the country. The centralised single-hub model used in this area (as well as the 10-week window the authors of the current study allowed for return, that is, longer than the 2 weeks allowed in past studies) may, in part, account for the high rate of completion.

Inequalities observed in CRC pathways generally also appear to be evident in completion of symptomatic FIT overall, as well as time to return.^22^ Recent work has shown that there is lower satisfaction with primary care consultations about symptomatic FIT in more socioeconomically deprived areas.^6^ Patients have reported that GP’s explanation of the test is often poor.^5^ Poor communication around the purpose and necessity of the test may be a barrier to completion that is particularly pertinent in the most socioeconomically deprived areas, where health literacy is often low.^23^

Lower return among male patients supports previous work that has shown that male patients have poorer engagement with the healthcare system generally.^24^ In the context of bowel cancer screening male patients also have lower uptake,^22^ possibly because of higher cancer fatalism and lower cancer knowledge.^25^ The current study found that younger patients were also less likely to return tests, possibly the result of more competing priorities (such as, employment and caring responsibilities)^26^ known to have an impact on health behaviours, or lower perceived (and actual) risk of CRC.^27^ Alternatively, it may be that older patients are more familiar with the process of completing FIT through past engagement with the national bowel cancer screening programmes.^28^

Ethnicity has long been associated with cancer inequalities. There is lower bowel cancer screening uptake,^29^ lower symptom awareness, and more advanced CRC at presentation among ethnic minority groups.^30,31^ The lower completion of symptomatic FIT among patients residing in the most ethnically diverse areas may be the result of cultural beliefs about cancer,^32^ shame, and embarrassment around cancer diagnosis and investigations.^33^

### Implications for research and practice

The demonstration of sociodemographic disparities in test return suggests research is urgently required to understand the barriers to and facilitators of symptomatic FIT completion and return. Future work should focus on groups for whom inequality has been evidenced and empirical research should be augmented with collaborative work alongside community stakeholders to develop strategies that aim to improve test return (such as, greater explanation of test purpose and process within GP consultations, or alternative information formats). Specifically, given increasing concern around rising rates of early-onset CRC^34^ and lower return of symptomatic FIT among younger patients, demonstrated in the current analysis, future work should focus on barriers to return experienced by those aged <50 years. Further work examining geographical variation in return would also be of value to understand how place interacts with sociodemographic factors and local FIT pathways to influence test return.

Looking beyond uptake, there is also a need to better understand whether, and if so, how, these inequalities have an impact on CRC diagnosis and outcomes, and, in particular, whether they exacerbate inequalities already evident in the UK.^12–15^ There is increasing interest internationally in the role and potential of FIT in the symptomatic pathway,^35^ with sociodemographic inequalities in CRC incidence and outcomes also reported in other European countries in which there has been interest in FIT.^36–38^ This suggests that these findings have relevance beyond the UK and that countries considering the implementation of FIT should ensure that robust systems are in place to monitor test completion overall and among population subgroups. Given the high return rate to this hub (which centrally distributes kits), evaluation of optimal methods for FIT distribution would be worthwhile.

In conclusion, overall return of symptomatic FIT was high and timely. However, sociodemographic patterning of (non-)return was both evident and, in relation to the potential exacerbation of CRC inequalities, concerning. It is vital that return is appropriately measured, barriers to (timely) completion identified, and interventions codeveloped with relevant communities to ensure that FIT does not introduce, or exacerbate, disadvantage for specific patient groups.

## Data

These data are not publicly available.
